# Rescuing Informed Consent: How the new “Key Information” and “Reasonable Person” Provisions in the Revised U.S. Common Rule open the door to long Overdue Informed Consent Disclosure Improvements and why we need to walk Through that door

**DOI:** 10.1007/s11948-019-00170-8

**Published:** 2019-12-23

**Authors:** Mark Yarborough

**Affiliations:** Bioethics Program, University of California Davis Health, 4150 V Street, Suite G100, Sacramento, CA 95817 USA

**Keywords:** Informed consent, Key information, Reasonable person standard, Research ethics, Risk–benefit assessment, Preclinical research, Social value of research, Nuremberg code

## Abstract

There is substantial published evidence showing that countless people enroll each year in ethically deficient clinical trials. Many of the trials are problematic because the quality of the science used to justify their launch may not be sufficiently vetted while many other trials may lack requisite social value. This poses the question: why do people volunteer for them? The answer resides in large part in the fact that informed consent practices have historically masked, rather than disclosed, the information that would alert research candidates to the ethically problematic nature of the trials. The “reasonable person” and “key information” provisions in the revised US Common Rule create the opportunity to correct this historical shortcoming. Two sources are employed to shed light on what the “key information” is that should be disclosed to a “reasonable person”: the original disclosure aims of the Nuremberg Code, as well as an extensive body of meta-research evidence. Those sources jointly support a range of new disclosures in the informed consent process that would unmask the heretofore undisclosed information. The resulting proposed new disclosures pertain to the overall success prospects of clinical trials, the quality of the prior research that both forms the basis of clinical trials and informs assessment of their risks and benefits, the potential social value of clinical trials, and the commercial purposes of clinical trials.

## Introduction

There is substantial evidence showing that thousands of people every year unwittingly volunteer for ethically suspect clinical trials. Some of the evidence pertains to preclinical studies used to justify the launch of early phase clinical trials. As the review below makes clear, more often than one might care to admit, the trials are launched on the basis of false positive and other problematic findings, showing that people are volunteering for studies that may lack a body of reliable science needed to affirm that the benefits of a trial are reasonable in relation to its risks. Amply documented problems about many later-stage industry-sponsored trials, also reviewed below, show how their undisclosed commercial aims can render them devoid of requisite social value. The result of this is that people volunteer for such trials despite the very real chance that a trial’s design could produce biased results that may cast what in reality is an inferior drug as a superior one. The evidence also shows how potentially misattributed superiority supports marketing efforts that can boost market share and consumer prices. Any resulting increased costs and clinical inferiority can then get passed along to the very volunteers who helped make the suspect trials possible in the first place.

There are multiple contributors to the troubling evidence referenced above. Each will require several reform efforts if the incidence of the problems that the evidence highlights are to be reduced. This paper cannot describe, let alone address, all the needed reforms. So, it will focus instead on just one of the avenues among the plethora of needed remedies: the disclosure component of the informed consent process for clinical trials. To appreciate this focus, readers need only ask themselves what all of the clinical trials plagued by the above problems share in common. Two things come to mind. One is the fact that hundreds of thousands of people volunteer to enter the ethically suspect trials. The other is that Research Ethics Committees (REC) routinely approve them. Thus, something must be askew both in the REC approved informed consent processes and the REC trial approvals themselves. This essay is devoted to strengthening the informed consent process. Elsewhere I address ways to strengthen REC trial review processes in order to better flag and thus help prevent the conduct of ethically suspect trials in the first place (Yarborough 2020).

Looking to the informed consent process, the evidence examined below will make clear that it is not flagging critical information that people need if they are to be able to make informed decisions. I will explore why this is so. I will argue that the reasons, ironically, reside in the very regulations, such as the “Basic Elements” of informed consent found in the Common Rule in the United States, that have stipulated informed consent disclosures. Though REC compliance with such regulations have assured “regulation compliant” informed consent processes, I will show that it has simultaneously suppressed disclosures that would have alerted research candidates^1^ to features of clinical trials that would likely have given many, and in some circumstances most, of them pause about volunteering for a given clinical trial. Once I make this case, I turn to the challenge of achieving greater transparency, through fulsome disclosures in the informed consent process, about evidence that touches on a range of matters clearly relevant to the decisions of research candidates.^2^

The proposed new disclosures are supported by recent and welcome changes to the Common Rule’s informed consent requirements. That Rule now states that research candidates or their legal representatives “must be provided with the information that a *reasonable person* would want to have,” viz., the “key information that is most likely to assist [someone] in understanding the reasons why one might *or might not* want to participate in the research.” [(see (4) and (5) (i), Box 1, emphasis added] One should note, though, that the Common Rule revisions do not stipulate what that key information is nor how it can differ from the “basic elements” of informed consent [See (6) (b) in Box 1].

**Box 1:** The common rule informed consent basics (§§ 46.116)

(4) The prospective subject or the legally authorized representative must be provided with the information that a reasonable person would want to have in order to make an informed decision about whether to participate, and an opportunity to discuss that information
(5) (i) Informed consent must begin with a concise and focused presentation of the key information that is most likely to assist a prospective subject or legally authorized representative in understanding the reasons why one might or might not want to participate in the research. This part of the informed consent must be organized and presented in a way that facilitates comprehension
(6) (b) Basic elements of informed consent:
(1) A statement that the study involves research, an explanation of the purposes of the research, … expected duration of the subject's participation, a description of the procedures to be followed, and identification of any procedures which are experimental;
(2) A description of any reasonably foreseeable risks or discomforts to the subject;
(3) A description of any benefits to the subject or to others which may reasonably be expected from the research;
(4) A disclosure of appropriate alternative procedures or courses of treatment, if any, that might be advantageous to the subject;
(5) A statement describing the extent, if any, to which confidentiality of records identifying the subject will be maintained;
(6) For …more than minimal risk, an explanation as to whether any compensation and an explanation as to whether any medical treatments are available if injury occurs and, if so, what they consist of, or where further information may be obtained;
(7) An explanation of whom to contact [about] questions about the research and … research-related injur[ies]; and
(8) A statement that participation is voluntary, refusal to participate will involve no penalty or loss of benefits to which the subject is otherwise entitled, and the subject may discontinue participation at any time without penalty or loss of benefits to which the subject is otherwise entitled
…

Rather than wait for future regulatory guidance about what that key information is, legal and bioethics scholar Nancy King has noted that the revisions create the opportunity for RECs to take some initiatives themselves and use the “key information” provision to “[better protect] the rights and interests of research subjects” (King 2019). Empirical research, particularly with the public and past research participants, has been widely endorsed as one method for improving disclosures because it can identify what these critical constituents think should count as key information (Kraft et al. 2017; King 2019; Dresser 2019). I concur that such research can reveal a lot about what information future research candidates would consider key to their decisionmaking.

While waiting for more empirical work of this nature to be conducted, there are two other sources one can look to immediately to help identify what should be considered key information. One is the Nuremberg Code and the other is already published research conducted by the meta-research (Ioannidis 2018), i.e., research about research, community. In what follows I draw from both of these sources to show why traditional regulation-compliant disclosures have effectively concealed what should now be regarded as “key information” going forward and what, specifically, some of the new disclosures should be.

Before turning to these matters, though, there is a proverbial elephant in the room that needs attending to. Many readers’ responses to the above examples, as well as to the assessment of the evidence to follow, may likely be along the lines of, “if there are so many ethically deficient studies, they should never be permitted to launch in the first place. Rather than worry about informed consent, we need to do a better job of weeding out the trials in the first place.” The author concurs both that clinical trials ought not launch in the absence of a careful determination that there is sufficient reliable scientific evidence that establishes that its benefits are proportional to its risks and that one ought not expend a research volunteer’s trust and good will in clinical trials that are in pursuit of deceptively gained profits. The evidence below, though, shows that the fact of the matter is that RECs all around the globe are currently approving such types of studies.

This is unlikely to change anytime soon for several reasons. For example, strengthening a REC’s ability to determine reasonable ratios between risks and benefits in early trials would require changes in the conduct and reporting of preclinical studies. RECs uncovering possible commercial abuses in later trials on the other hand would require information from sponsors, such as internal communications about marketing strategies and protocol designs, that sponsors would likely refuse to disclose in the absence of a legal requirement to do so. Finally, the government regulations driving key REC practices about weighing benefits and risks and assessing social value that result in REC approvals are unlikely to change any time soon. Witness how long and contentious the process to change the U.S. Common Rule has been. Thus, while RECs wait for their governing regulations to be amended, the culture of preclinical research and reporting to improve, or corporate behavior to become more socially responsible, thousands of research participants every year will continue to be asked to volunteer for dubious trials. So, if there are to be any immediate reforms, they must be driven by changes that RECs can unilaterally impose. And the new informed consent provisions in the revised Common Rule have opened the door for such unilateral changes: they can be used to strengthen REC oversight of informed consent disclosures. No doubt, as mentioned previously, such reforms will not cure all that ails far too many clinical trials. That ought not deter us, though, from pursuing ethically sound reforms when possible.

I endorse informed consent disclosures as a needed avenue of reform fully recognizing that informed consent itself is a flawed tool and new disclosures will not remove all the flaws that people have long fretted over. Some of those flaws no doubt contributed to the informed consent revisions in the Common Rule in the first place. Nor should one overestimate the power of information disclosure itself (Schneider 2005). These limitations notwithstanding, the Common Rule revisions still present a critical opportunity to improve informed consent.

As I hope to make clear below, they can help us to do a better job than has been done up to now in figuring out the disclosure side of the informed consent equation, which would both ethically strengthen informed consent and permit us to utilize the transparency it would promote as one among the many reforms that will be needed to better assure the ethical conduct of clinical trials. Let us now turn to the Common Rule to see how it has traditionally treated the disclosure side of informed consent, as well as how the new “key information” and “reasonable person” provisions have opened the door for more robust, ethically sound disclosures going forward.

## What the Nuremberg Code can Teach us About what Should Count as “Key Information” that “Reasonable” People Deserve to be told

Nothing is more fundamental to the ethical conduct of clinical trials than the informed consent of research participants. The Nuremberg Code and every subsequent major international statement about ethics and medical research enshrine this role. Furthermore, government regulations and guidelines, such as the “Common Rule” in the United States (HHS.gov 2017), the “Informed Consent Guidance for Applicants” of the European Commission Research Directorate (Directorate-General and Directorate), and the “Requirements for Informed Consent Documents” of Health Canada (Research Ethics Board 2014), codify it. Significant human and financial resources are invested worldwide among researchers and REC members and staff to comply with these regulatory directives.

How is it, then, that despite this investment, regulation compliant informed consent processes up to now have failed to adequately inform research volunteers? Most assuredly, it is not due to nefarious people serving on RECs. Rather, the way that the Common Rule and similar regulations in other countries codified informed consent disclosures is the principle culprit in my view. Box 1 contains the information relevant to informed consent disclosures in the revised Common Rule. Readers should note that the “Basic Elements” (item 6.b) remain unchanged in the newly revised rule and it is these provisions that have driven US REC practices for decades. A review of regulations about informed consent disclosures found in Box 2 from the Informed Consent Guidance for Applicants of the European Commission Research Directorate and the Requirements for Informed Consent Documents of Health Canada reveal a strong similarity to the Common Rule “Basic Elements” and thus drive similar REC disclosure practices in those jurisdictions as well.

**Box 2:** Directorate-general, European Commission guidance for applicants informed consent

General information:
A statement that the study involves research subjects and an **explanation of the purposes** of the research
The **expected duration** of the subject's participation
A **description of the procedures** to be followed/ of the **medicine** that is going to be tested, and an identification of any procedures which are experimental
A statement that participation is **voluntary**
Information about who is organising and funding the research
A description of any reasonably **foreseeable risk, discomfort or disadvantages**
A description of any **benefits to the subject or to others** which may reasonably be expected from the research avoiding inappropriate expectations
A disclosure of appropriate **alternative procedures for treatment/diagnosis** if any, that might be advantageous to the subject. (*emphasis in original*)
**Requirements for Informed Consent Documents of Health Canada** (*relevant portions only*)
Introductory information
Title of research project
The identity of the researcher(s)
The purpose of the research—Why do the study? Provide a brief description of the purpose of the study
That the individual is invited to participate in research
The basis for inviting the individual to take part. (Include information on any criteria under which prospective participants would be excluded from participation)
That the individual's participation in the research is voluntary and that the individual may refuse to participate or may withdraw from the study, at any time, without penalty or loss of benefits to which he/she is otherwise entitled
The purpose of the research. Be sure that the description of the purpose provided in the consent documents is consistent with the purpose as described in the protocol
What will the participant be asked to do?
Describe the research procedures that the participant will be involved in
State the expected duration of the participant's participation in the research
Risks/benefits
The reasonably foreseeable risks, harms, or inconveniences to the participant
The reasonably expected benefits. When there is no direct benefit to the participant, the participant should be made aware of this
If blood is taken, a statement noting the possibility of bruising or swelling while giving blood, or other possible discomforts at the site where blood is drawn. Indicate that there may be minimal chance of infection and that discomforts experienced will be brief and transient

What one sees in all these instructions is a parsimony borne of a focus of the central elements of informed consent that relate to the research to be undertaken on how participation in it primarily physically impacts volunteers. They emphasize the body, particularly what is to be done to it and how it might be affected, which results in informed consent practices that conceal key information from research candidates. New disclosures are needed if one hopes to avoid the detriment this parsimony causes to research candidates.

Support for more robust disclosures resides in relevant portions of the Nuremberg Code (see Box 3) that speak directly to the stated goal of Section (5) (i) in the revised Common Rule of assuring that research candidates understand “the reasons why [they] might or might not want to participate in [a clinical trial].” Those portions, found in the bolded text in Box 3, stipulate what research candidates need to know and why. First, they state that research candidates must have sufficient information about “the elements” of the research in question. If the disclosed information does not enable an “understanding and enlightened decision,” then information about those elements is insufficient. Both the “nature” and “purpose” of the research, its “hazards,” and the “effects,” not just to one’s health but to one’s “person,” are also stipulated disclosures. Only this extent of disclosure renders research candidates capable of understanding the implications of granting permission to be used in research.

**Box 3:** The Nuremberg code on informed consent

The voluntary consent of the human subject is **absolutely essential**
This means that the person involved should have legal capacity to give consent; should be so situated as to be able to exercise free power of choice, without the intervention of any element of force, fraud, deceit, duress, over-reaching, or other ulterior form of constraint or coercion; and should have **sufficient knowledge and comprehension** of the **elements of the subject matter** involved, as to enable him to make **an understanding and enlightened decision**. This latter element requires that, before the acceptance of an affirmative decision by the experimental subject, there should be made known to him the **nature**, duration, and **purpose** of the experiment; the method and means by which it is to be conducted; all inconveniences and **hazards** reasonably to be expected; and the **effects upon his** health or **person**, which may possibly come from his participation in the experiment
(*emphasis added*)

To see how regulatory regimes’ required disclosures to date have been more parsimonious than these key portions of the Nuremberg Code intend, it helps to compare references to the “elements,” “purpose,” “hazards,” and “effects [to one’s] person” in the Nuremberg Code with the Common Rule’s “Basic Elements.” Several things stand out in the comparison. One sees that the reasonably foreseeable “hazards” and “effects” found in the Nuremberg Code have been replaced with “risks and discomforts.” While there is little difference between “hazard” and “risk,” “discomfort” is more delimiting than is “effect.” The Nuremberg Code’s “elements of the subject matter” of the research are not referenced, having been replaced instead with an explanation of “procedures.” Most notable is that the “Basic Elements” do not reference effects upon one’s “person.” Hence my statement above that regulatory treatment of the central elements of informed consent work to confine disclosures to how research participation primarily physically affects volunteers.

The parsimony results in the routine suppressing of information about four important features of research that evidence discussed below pertains to: the overall success prospects of clinical trials, the quality of the prior research that both establishes the scientific basis of clinical trials and informs assessment of their risks and benefits, the potential social value of clinical trials, and the commercial purposes of clinical trials. All of these features, I contend, are “elements” of the research that research candidates have an interest in knowing and thus represent “key information that is most likely to assist [people] in understanding the reasons why [they] might or might not want to participate in the research.” To establish the relevance of all four of these features of clinical trials to research candidates’ decisionmaking, it is important to note some very basic considerations about clinical research, so that is where we now turn.

## Remembering Some Basic Considerations About Clinical Research

Clinical trials are a discovery endeavor whose key components include a question to be investigated, a group of investigators, those who sponsor the investigation, and those who are the subjects of it. In short, it is an activity that uses people, usually at some risk to themselves, in order to acquire knowledge to benefit others (Miller and Brody 2003). The requirement for informed consent arises because research candidates, as people due basic respect, need to know what they will be used for and how such use might impact them. This helps explain why the Nuremberg Code deems such consent “absolutely essential.”

In short, research candidates need to know enough about the research so that they can understand how participating in it might affect their rights, interests, and welfare, as well as whether or not they want to partner with investigators and sponsors, both in answering the questions that trials are meant to shed light on and pursuing the goals the answers are meant to serve. Informed consent processes that fail to convey information about any of these features of research surely conceals some of the “key information that is most likely to assist a prospective subject or legally authorized representative in understanding the reasons why one might *or might not* want to participate in the research.” (emphasis added).

In addition, it is also important to note the social and ethical placement of clinical trials, found in the background frame of biomedical research (Tversky and Kahneman 1981; Charuvastra and Marder 2008). That frame situates research as a noble pursuit of discoveries meant to improve people’s lives, often in profound ways. The marketing efforts of pharmaceutical and device manufacturers, as well as hospitals (London and Kimmelman 2018; Yarborough et al. 2019), regularly reinforce this message. This frame fuels powerful biases such as unwarranted optimism and overestimation of personal benefit (Harrop et al. 2016; Nurgat et al. 2005; Reijula et al. 2017; Jansen et al. 2011) that research candidates can bring with them to the informed consent process, highlighting the need for RECs to provide research candidates with debiasing information (Chwang 2016) during the informed consent process.

The very proffer of an informed consent form is also an implicit endorsement of the scientific and social merit of a clinical study. Approaching research candidates about trials under REC jurisdiction attests that a REC has made an independent and careful assessment that establishes that several critical thresholds have been met: there is a body of sound evidence that establishes “justified belief” in a drug or device’s promise (Kimmelman and Henderson 2015), the nature of the promise is such that it is proportional to all the potential risks entailed by its pursuit, and the trial is an instance of disinterested science capable of producing unbiased knowledge. As shown below, there is often evidence that calls the accuracy of both this endorsement and the background frame into question, evidence critical to research candidates being able to understand what others are wanting to use them for, and why, which presently falls by the wayside due to the historically parsimonious disclosures in informed consent processes. With these basic considerations about clinical research and informed consent in mind, let us now turn to the previously referenced evidence to understand why it is key information that needs to be disclosed in the informed consent process.

## Fleshing out what the “Key Information” is that a “Reasonable Person” Would want to Know

### The need for Disclosures About the Overall Prospects of Research

The stipulated disclosures found in the informed consent instructions of the referenced EU, Canadian, and U.S. documents do not require disclosures about the overall prospects for the success of trials research candidates are being asked to help make possible, even though it is these prospects that establish proportionality between the benefits to society likely to be produced by trials with the risks they will impose on research participants. This results in informed consent processes that eschew information that actually speaks about those prospects.

Evidence that sheds light on those prospects is readily available and much of it is likely at drastic odds with the motivations that drive many, perhaps even most, people to volunteer for clinical trials. Presumably people volunteer because they want to help shed light on health issues of interest to themselves or others. What does the evidence show the prospects are for generating new knowledge capable of shedding additional light on a drug or device? Ample evidence about the prospects for clinical trials generating new knowledge make two things clear. First, the most likely knowledge to be learned from a trial is that the new drug or device does not work as well as hoped. For example, the failure rate of Phase I trials exceed 90% on average, while about half of Phase II and III clinical trials fail because of lack of efficacy, and another quarter fail because of safety concerns (Harrison 2016). Second, the chances are high that much, and possibly even all, of this knowledge, and thus the benefit gained by conducting the trial, will not be shared with others because the results very likely will never be published in full or possibly even in part (Riveros et al. 2013). Therefore, the “light” of new knowledge that volunteers are motivated to help shed all too frequently never is allowed to shine.

My argument in favor of disclosures about the prospects for a trial’s success is not made in order to suggest that high failure rates in terms of eventual approval of new drugs or devices render a trial somehow unimportant and thus not worth the contributions of research participants. Instead, the point is simply that since disclosures about the prospects for risks in a study are already made and since informed consent processes are supposed to help research candidates weigh possible benefits and risks themselves as they ponder whether to be exposed to risks or inconvenience, it makes little sense not to disclose information about the prospects for benefits that is comparable to the disclosures made about the prospects for risks. Not doing so is tantamount to saying that information about the prospects for risks is key information but information about the prospects for benefits is not. This imbalance in disclosures results in many, perhaps even most, research candidates entering trials not realizing how modest the prospects for success, e.g., increased knowledge, may be, given that the partial, let alone full, results made possible by their participation may never be published. Nor would they know the high likelihood that trials will not result in either advancement to the next phase of research or ultimate approval of the investigational drug or device, which is also information that many research candidates may deem relevant to their deliberations.

### The need for Disclosures About Salient Features of Prior Research

Given the importance of proportionality between risks and benefits, research candidates also need disclosures about the prior research relied upon in assessing proportionality. This is especially so for early human trials. After all, the predictive value of informed consent disclosures about risks and benefits is only as good as the prior research that was used to determine them.

Can research candidates have confidence that the clinical trials they are being asked to participate in are based on solid science that establishes not that people think a new drug or device is going to work, because this obviously can never be established in advance, but that there is instead at least a rational prospect that it conceivably might? Absent this rational prospect, there can be no justification for studying either the safety or efficacy of a new drug or device in early trials of it. The evidence suggests such confidence is often ill-advised, but most research candidates will not know this if informed consent processes suppress relevant information about such evidence.

For starters, preclinical studies frequently produce false, as opposed to true, findings. This is due to the fact that most preclinical studies are really hypothesis generating, not hypothesis confirming, studies (Kimmelman et al. 2014). Thus, what can appear to be promising results may in fact be false findings that forecast the likely failure of a trial. This matters because trials can launch in the absence of the follow-up preclinical studies that would be needed to determine if the initial positive result suggesting the potential for benefit was a true positive finding or not. The failure to conduct confirmatory preclinical studies is but one of many well-documented concerns in preclinical research. Others include underpowered studies (Ioannidis 2005), as well as concerns about internal (Crossley et al. 2008), construct (Hackam and Redelmeier 2006), and external validity (van der Worp et al. 2010). Such evidence might shake the confidence of research candidates that the benefits of a study are in line with its risks so it calls into question current informed consent processes that never broach any of these well-known problems in the quality of preclinical research.

In addition to the quality concerns ascribed to preclinical research generally, additional problems with particular types of preclinical studies raise concerns that pertain in turn to specific types of clinical trials. Research candidates in cancer trials for example, would likely value disclosures pertaining to whether mislabeled cell lines were possibly used in preclinical studies since they can lead to erroneously launched clinical trials. Preclinical studies meant to investigate esophageal cancer, for example, may have actually been conducted on cell lines from a different type of cancer (Tadich 2016; Boonstra et al. 2010).^3^ Evidence about external validity for certain classes of research can prove equally problematic (Pound and Ritskes-Hoitinga 2018). For instance, no drugs shown effective in preclinical studies in rodents to combat either Alzheimer’s disease ( Sarawitz 2016) or stroke (Dirnagl 2016) have ever shown any success in people. Surely some research candidates for such trials would find such information pertinent to their deliberations (Gotzsche 2014).

Similar concerns can apply to preclinical research for specific Phase I trials. Consider people who volunteer for them. Even though they are informed that the trials are meant either primarily or exclusively to study safety, one knows from the previously referenced research about unwarranted optimism and overestimation of personal benefit—a phenomenon that is commonly referred to as therapeutic misestimation (Horng and Grady 2003)—that many volunteers nevertheless believe that new drugs may help them.^4^ What, then, might they make of the fact, if it was disclosed to them, that regulatory approval agencies such as the U. S. Food and Drug Administration (FDA) may have only reviewed safety data from preclinical studies (FDA)? In other words, what would research candidates make of the fact that drugs can enter human trials without any preclinical data supporting efficacy? As regards preclinical evidence about new investigational drugs that are approved with both safety and efficacy data, those data may diverge significantly from patient expectations.^5^ For example, ALS patients may enter a Phase I trial with hopes for greater life-expectancy should the new drug be effective, yet the efficacy endpoints used in preclinical studies may not have looked at survival benefit at all, looking instead at matters such as gait (Hefferan et al. 2012).

Informed consent forms fully compliant with the disclosures historically required by regulations typically disclose no information related to any of this evidence, despite its clear relevance to decisions about joining a clinical trial. Regulations have not required disclosures about what is either known or unknown, either generally or specifically, about the prior research used to judge potential risks and anticipated benefits, nor why the information about that research is thought reliable enough for a REC to use (Kimmelman and Federico 2017). It is hard to square such non-disclosure practices with the weight of all the assembled evidence.

### The need for Disclosures About the Potential Social Value of Research

Turning now to later phase trials as opposed to early ones, a new set of disclosure issues pertaining to a trial’s importance emerge. A requisite amount of social value has long been recognized as an ethical requirement for the conduct of clinical trials (Emanuel et al. 2000). Informed consent processes typically devote little more than cursory information about this value for any given trial. Regulations can stipulate that there be a description of benefit to participants and/or “others,” but they do not require any disclosures about the strength of prospects for that benefit.

This is problematic when one recalls that the proffer of an informed consent form to a research candidate is an implicit endorsement that it is important that the trial get conducted. Such endorsements are strengthened by the background frame effect (Charuvastra and Marder 2008; Bubela et al. 2009; Caulfield 2018) that results from dominant hyped discourses that situate clinical trials in the pursuit of breakthrough discoveries that can cure diseases and improve the quality of lives (Caulfield 2018). Yet, basic facts about many later phase clinical trials reveal the poor fit this background frame has with them.^6^ Consider evidence pertaining to the various commercial purposes that drive countless clinical trials, and thus what research candidates are being asked to help make possible. These purposes rarely if ever get broached in informed consent processes. Evidence about two kinds of trials and the commercial aims behind large numbers of each illustrates why this is problematic.

The first type is known as head-to-head trials. They compare the effectiveness of competing drugs. Frequently, such trials are “non-inferiority” trials that study whether a new drug is “not significantly worse” than a currently available efficacious treatment (Palmas 2018). As researcher Walter Palmas who also writes about the ethics on non-inferiority trials describes, such trials, by design, tolerate some loss of efficacy compared to the current standard of care for the research candidates randomized to the test drug in hopes that this loss of efficacy will be offset by some improvement, such as greater tolerability, safety, or convenience. At times, “the clinical outcome of interest is severe, such as death,” highlighting both the ethical complexity such trials can have and the importance of their proper design (Palmas 2018).

When designed and conducted appropriately, such trials can have immense social value because they can tell us whether one of those drugs is actually substantially better than the other. Sadly, a recent study of head-to-head trials showed that most of the trials actually had negligible social value (Fiacco et al. 2015). The study consisted of a random review of 319 randomized clinical trials published in 2011 that were head-to-head comparisons of drugs and biologics. Most of them (82%) were funded by industry. Only 3 of the trials were deemed to be “truly antagonistic” comparisons that were capable of detecting actual greater social value. Nevertheless, the study found that all but 2 of the industry-sponsored trials reported favorable results that, as is discussed shortly, can be used to boost marketing efforts and future prescribing practices for undeserving drugs.

Seeding trials, which are another type of marketing study done to familiarize physicians with a drug, raise concerns similar to those of head-to-head trials. A recent study that looked at 194 drug trials, also published in 2011, in 5 leading medical journals documents this (Barbour et al. 2016). The study was carried out to examine whether trials might have been conducted in order to get physicians to prescribe a new drug. To see if this was so, the authors carried out a study to test their concerns about the frequency with which clinical trials get done in order to boost a drug’s marketability to physicians. They completed the study by looking at publications of trials that were suspicious for marketing purposes whose results were published in the *New England Journal of Medicine*, *The Lancet*, *Annals of Internal Medicine*, *PLOS Medicine*, and *BMJ*. They concluded that 41 of the 194 trials were found to be “suspected marketing.”

They did so because the suspected marketing studies tested drugs on patients from an average of 171 different geographical areas, whereas trials deemed not to be marketing trials recruited from just 13 different geographical areas. When one considers how much higher the number of prescribing physicians is who get exposed to the study drug in the trials suspected of being conducted for marketing purposes compared to the number of prescribing physicians in those studies not suspected of being done for marketing purposes, the true purpose behind the suspect clinical trials is apparent. Nor should one fail to note the fact that all the suspected marketing studies were industry funded.

Such evidence shows that people are being recruited into trials to advance the financial interests of the sponsors, since physicians who recruited their patients into the trials would be more likely to prescribe the study medication to their future patients after completion of the trial. This impact is greatly compounded when one considers that other physicians will read about the studies in prestigious medical journals and may change their prescribing practices as a result. One reason they may be more likely to do so is because the articles are often tainted by what is known as “attributional bias,” wherein the involvement of academic researchers is highlighted in the publication while the role of the industry sponsor gets downplayed (Matheson 2017). Another is that the studies are written about in such a way that the marketing influences on them are “difficult to identify by the average [physician] reader” (Barbour et al. 2016).

Current informed consent processes facilitate all of this by disclosing partial information, such as a “study is being conducted in order to see how well a new drug works,” while simultaneously concealing the overriding purpose of the study: produce data for high-profile reports that will be used to influence physician prescribing practices to choose one competing and potentially inferior drug over another. This concern is borne out by a recent study that looked at the informed consent forms of clinical trials designed to assess non-inferiority hypotheses in antibiotic trials comparing two competing drugs against each other. The study, which reviewed informed consent forms for 78 randomized trials that enrolled almost 40,000 volunteers, found that almost all of the consent forms failed to accurately describe the purposes of the study (Doshi et al. 2017).^7^

All of this evidence is deeply troubling. At a minimum, hundreds of thousands of people (Fiacco et al. 2015) volunteered for all of these trials. These large numbers of volunteers are due in large part, no doubt, to two obvious features of the trials’ informed consent processes. First, the regulation-compliant processes failed to flag any information suggesting suspect design of the trials that would be needed to counter other information in the informed consent forms that implicitly endorses the need to conduct them. Nor should one assume that volunteers were informed that, by volunteering for the trial, they would be contributing to a sponsor’s aim to get study results published in high-impact journals where they would have an outsized influence on future clinical guidelines that will drive prescribing practices to the possibly inferior drug (Fiacco et al. 2015). Furthermore, it should be noted that non-disclosure about these kinds of commercial considerations is at odds with at least one published study showing that research participants want to be told this kind of information (Cook and Hoas 2011).

All this evidence begs for justification for continuing to use informed consent processes that sanction silence about it. Such justification would have to show that it is permissible to use research volunteers to pursue undisclosed commercial purposes even when this might lead, for example, to higher prescription prices for a non-inferior or, depending on the extent of flaws in trial design, a possibly inferior drug. It would also have to explain why these commercial purposes and potential personal cost and health outcomes are not among the “elements” of research that research candidates have an interest in knowing. Finally, it would need to show that partial, and arguably quite misleading, disclosures about a trial’s purpose that mask the major reasons why studies are conducted are somehow considered capable of helping research candidates make “understanding and enlightened” decisions as they ponder why they “might or might not want to participate” in the trial.

### Counterarguments

No doubt some will raise questions about the expanded disclosures advocated here on the basis of the aforementioned longstanding concerns about the informed consent process itself. For instance, why call for more disclosures when no one disputes that informed consent forms are already too long and opaque? Others may question why disclosures about the matters highlighted here are thought capable of producing the desired effects, given what was mentioned before about what is known about the limited effectiveness of disclosure in general (Schneider 2005).

Such concerns mainly involve worries about the effectiveness of informed consent itself rather than the specific disclosures presented here for reforming it. So, due to space considerations, they will not be addressed in depth. Instead, readers can note that the informed consent changes being advocated need not result in voluminous disclosures. For example, a disclosure for a Phase I trial might read “Since this study is what is known as a Phase I study, there is only about a 5% chance that the drug will eventually be found to be effective.” A sample disclosure for an industry sponsored head-to-head trial suspicious for being done primarily for marketing purposes,^8^ should a REC decide to approve such a trial despite those suspicions, might read “This study is what is known as a head-to-head trial that compares two different drugs meant to treat the same condition. This study is also being paid for by the manufacturer of one of those two drugs. Often with these kinds of trials, the company paying for the study designs it in such a way that it will produce results suggesting that its drug may be preferable, even though the trial’s design may not be capable of establishing this. They do this to boost the appeal of their drug to physicians in hopes that they will start prescribing it rather than one of the other competing drugs. We do not know whether this particular study is like that because the company paying for the study would not provide our committee with the information it would need to determine whether or not the study was designed in a way to produce misleading results.”^9^

Readers should also entertain the possibility that, given the extent to which the information found in the advocated new disclosures is at odds with the background frame about research and the presuppositions that research candidates can bring with them to the consent process, these disclosures are likely to stand out in the informed consent process compared to other content in it. If so, the information might help to make headway against both therapeutic misconception (Appelbaum et al. 1982; Kimmelman 2007), which refers to the phenomenon of research participants failing to appreciate how research differs from clinical care, and previously referenced therapeutic misestimation, and thereby increase the effectiveness of informed consent.

There is a more germane concern about the proposed new disclosures that warrants a more fulsome response, which is that the informed consent process was never meant to endorse unlimited disclosures. That is why there is a threshold that needs to be met in order to trigger disclosures. For example, there is no need to disclose every conceivable risk that might occur during a clinical trial. Instead, thresholds of severity and/or prevalence often guide disclosure practices. Acknowledging the legitimacy of such thresholds, an explanation about why the recommended disclosures trigger them is in order.

Consider a hypothetical situation. What if hospitals did not check physician credentials, meaning that at times unqualified surgeons performed surgeries at hospitals? If a REC was reviewing a clinical trial that involved surgery, it would want to both guard against this risk and disclose it to research participants. Of course, in reality such possibilities are never actually disclosed by RECs because licensure and credentialing requirements render risks that impostors may find their way into the operating suite so remote that it would be silly to disclose such a rare occurrence. But the same assurances often cannot be made about the provenance/quality of prior research. Thus, the risk is far from remote that flawed prior research will be relied upon during the REC review process. Depending on the nature and severity of the flaws in the prior research and publications about it, harms similar in severity to those that unqualified clinicians can cause are possible.

To claim that prevalence of this risk falls below threshold triggers ignores the extensive evidence that exists about research quality. It would also mean that research candidates have no alternative other than to have blind faith that RECs can and will check the provenance of the research that was relied upon to assure that there is a proportionate risk/benefit ratio, even though such careful checking is currently outside the norm of most REC practices. This reveals genuine prospects that assessments about a trial’s value, risks, and benefits may be less reliable than research candidates would presume, which means that the research itself may be less valuable or more risky than appears, and that the risk/benefit ratios may be less favorable than a REC has determined. Such information is surely germane and thus should be counted within the “key information” needed to enable “understanding and enlightened decision[s].” The fact that RECs have not historically been specifically instructed by regulations to either inquire about or disclose such matters to research candidates does not trump the legitimate interests those candidates have in knowing about them.^10^

Readers may grant the above points but disagree about the need for additional disclosures since consent forms routinely state that there may be “unforeseeable risks” in the research. Hence, little is gained by the proposed disclosures. But the risks that the proposed disclosures are intended to mitigate are foreseeable rather than unforeseeable and disclosures about them to research candidates will help them in their risk/benefit assessments. Simply telling people that there may be unspecified, unforeseeable risks masks the nature and extent of them, rendering any weighing of benefits against risks incomplete.

A final counterargument claims that the proposals set forth here are far too modest. This counterargument is suggested by prior commentaries concerned with the proverbial elephant in the room referenced at the outset. It contends that, rather than disclosure, what is needed is an end to REC approval in the first place of trials tainted by some of the ethical problems reviewed here. For example, in order to avoid the risks and waste posed by unnecessarily duplicative clinical trials, bioethics scholar Julian Savulescu and colleagues proposed in 1996 that RECs require investigators to conduct systematic reviews in order to determine whether or not there were prior studies that had already answered the questions to be investigated by a proposed trial (Savulescu et al.^1996^). Since then, bioethics and preclinical research expert Jonathan Kimmelman and colleagues have recommended that RECs “condition approval of any trial delivering putatively active drug doses on positive preclinical confirmatory studies” (Kimmelman et al. 2014). Most recently, philosopher Kirstin Borgerson has made an even more sweeping proposal by calling for RECs to only approve studies that exhibit “scientific integrity” (Borgerson 2014).

There is no doubt that these other remedies would prevent many of the problems highlighted here but, as I mentioned at the outset, they will remain in abeyance until such time as deep-seated practices, such as unreliable preclinical research practices or unimportant industry-sponsored trials, are reduced. Resolution of these matters is a massive undertaking the research community and governments have largely resisted to date. So long as this persists, and so long as current informed consent disclosures remain unchanged, then thousands upon thousands of people will continue to volunteer for clinical trials oblivious to the existence of the kinds of problems reviewed here that may be tainting any given trial. Reformed informed consent practices, that start with ethically adequate disclosures, is really the only viable option at this moment for guarding their interests.

The Common Rule “key information” and “reasonable person” informed consent revisions are the ideal catalyst for pursuing that option. The new disclosure standards could produce much needed transparency about the potential for the range of ethical deficiencies discussed here. And RECs are well-positioned to create this transparency. They are capable of, comfortable with, and good at gathering information, information that could subsequently be disseminated to research candidates through the re-imagined informed consent process envisioned in the Common Rule changes. So, persuading RECs to make to research candidates the disclosures advocated here should be an achievable goal.

The potential power of the transparency that would result from the new disclosures will improve the informed consent process by better aligning it with the original disclosure aims of the Nuremburg Code. It will also provide critical information to many REC members and compliance officials that the informed consent process is entrusted to. They likely are not sufficiently familiar with the evidence reviewed here and thus do not appreciate its effect on the trials they have historically been approving. This also means that they likely have not sufficiently considered to date the extent to which most research participants would value knowing this information about the trials they are volunteering for. No other explanation for why people would approve or enroll in such problematic trials seems plausible. Though empirical study would ultimately need to determine the scope and impact of the increased transparency that would result from the proposed new disclosures, it could very well improve current troubling portions of the clinical trials landscape. Again, the Common Rule revisions have set the stage for instigating it.^11^

It must be noted, however, that transparency is no magic wand. Concomitant, or even preparatory, steps will be needed for it to achieve its potential. Probably the most critical is REC member education so that they can become familiar with the range and extent of issues discussed and alluded to in this essay and, more importantly, the ethical implications of them. This will be a large undertaking but at least part of the infrastructure needed to complete it is already in place. Many RECs have education and training requirements for their staff and members and these new topics could be integrated into them.

## Conclusion

If informed consent is “absolutely essential” and if it requires “key information” that can differ from the basic elements of informed consent stipulated in the Common Rule and other research regulations, then new standard disclosures are surely in order. Some of these new disclosures should be made for virtually every clinical trial. For example, how does one justify continuing the current parsimonious disclosures about compelling evidence related to key features of clinical research, such as the prospects for their success or the prospects that what is learned in them will be sufficiently disseminated? Can research candidates reasonably weigh the risks and inconveniences entailed in research when such basic information about the most likely outcomes of a study is withheld from them? Other new disclosures should become standard for specific types of trials. For example, can research candidates for early trials make good decisions when the extent of uncertainty about the reliability of REC risk/benefit assessments is not made known to them (Yarborough et al. 2018)? Can research candidates for some later trials make good decisions if information about the potential social value of trials is treated as immaterial to their decisions? Do they really not need the truth about the basic reasons why sponsors want to conduct trials on them? Rather than continued use of informed consent processes that mask these categories of critical information, what is needed are ones that assure greater transparency about them. The avenue for that transparency, viz., the new informed consent provisions in the Common Rule revisions, is before us if RECs can muster the resolve to embark upon it. If they do, then at least one of the tools among the many that are needed to improve clinical research would begin to be employed. Even more importantly, research candidates would finally start to be treated with the level of respect that is their due as persons.

## Footnotes

1 I use the phrase “research candidate” to refer to people considering participating in a clinical trial. I use the more familiar term “volunteer” or phrase “research participant” to designate the people who actually enroll in trials.

2 The argument below about why our informed consent practices need to be altered draws heavily from empirical evidence, some of which was also drawn from in other essays (Yarborough 2017, 2020). Thus, readers may note some redundancies among these publications in the descriptions of the empirical evidence.

3 NIH now has efforts underway to reduce the incidence of research with mislabeled cell lines (Barrett 2013), but these efforts have limited reach and effectiveness, meaning that the problem will persist for some considerable time.

4 It should be noted that commentators disagree about the ethical significance of this misestimation. Clinician and bioethics researcher Scott Kim and his colleagues argue that statements of research participants that are cast as evidence of misestimation or misconception are best understood instead as statements about hope (Kim et al. 2009, 2016) whereas bioethics scholar Jodi Halpern and her colleagues argue that misestimations remain ethically problematic so long as informed consent processes do not permit research candidates to appreciate how trial participation will affect their lives, which most processes currently do not. (Halpern et al. 2019).

5 Personal communication, Nanette Joyce, DO.

6 Here the author does not mean to ignore the existence of breakthroughs and their significance. They do indeed occur and richly deserve celebration. Rather, the intention is to highlight that they have an oversized presence in the background frame for research.

7 The study employed two types of informed consent reviewers: research methodologists and patients. The methodologist reviewers found that only 1 out of the 50 consent forms reviewed accurately conveyed a study purpose while the patient reviewers found that only 11 of the 50 did.

8 No doubt many readers will question why the author would entertain a REC approving a study that is not known to have sufficient social value in the first place. This is a fair question the answer to which involves both ethical and logistical considerations that relate to REC vetting of studies themselves, a topic I explore elsewhere (Yarborough 2020).

9 Similarly, for non-inferiority trials, Palmas has recommended the following: “Institutional review boards (IRB) should require that very clear and specific language be used in explaining the risks of participation in a non-inferiority trial. There should be a statement describing, in very simple terms, the specific additional risks a participant may be exposed to. If that risk is death, the consent form should unequivocally state so” (Palmas 2018). As noted in the main body, none of these disclosures is inordinately long. Of course, deciding upon the final wording for new disclosures will prove to be an iterative process. RECs and the institutions they serve will have to agree on the need, extent, and merit of new disclosures; RECs will have to develop policies and instructions so that applicants can comply with any new required disclosures; and thought will be required to discover language to be included in informed consent forms that is succinct, comprehensible, and accurate.

10 The proverbial elephant in the room is raising its head once again. For starters, it is not at all clear that applicants would be able to provide all of the information needed for a protocol review that would be able to spot the problems of interest. This will leave RECs at an impasse. They could choose to disapprove applications missing the information needed for a more thorough review or they could choose to disclose what remains uncertain in the review to research candidates. This latter option is the one I implicitly endorse, not because I think it is necessarily ethically sufficient. Rather, I think there are times when it could be – think First-in-Human studies with seriously ill patients for whom there is no effective treatment. Different RECs could decide differently about whether there is a reasonable ratio between anticipated benefits and risks because there is very limited (or very limited relevant) preclinical research to go on but great need for research. I also prefer the latter option because I think it at least represents an improvement over the status quo. Too often now there is inadequate REC review with inadequate disclosures to research candidates. A trial conducted on the basis of adequate disclosures about the potential problems of inadequately vetted protocols strikes me as an initial step in the right direction.

11 If RECs adopted the proposed changes, they would no doubt encounter fierce resistance by both sponsors and investigators. This does not negate the existence, though, of the current deficiencies that create the need for the proposed changes. Nor do such challenges negate the fact that RECs must protect research candidates’ legal rights to grant valid informed consent, which, if the analysis found here is correct, they currently fail to do.
